# Pemphigus Vulgaris Confined to the Gingiva: A Case Report

**DOI:** 10.1155/2011/207153

**Published:** 2011-05-11

**Authors:** Mitsuhiro Ohta, Seiko Osawa, Hiroyasu Endo, Kayo Kuyama, Hirotsugu Yamamoto, Takanori Ito

**Affiliations:** ^1^Department of Oral Diagnostics, Nihon University School of Dentistry at Matsudo, 2-870-1 Sakae-Cho Nishi, Matsudo 271-8587, Japan; ^2^Research Institute of Oral Science, Nihon University, School of Dentistry at Matsudo, 2-870-1 Sakae-Cho Nishi, Matsudo 271-8587, Japan; ^3^Department of Periodontology, Nihon University School of Dentistry at Matsudo, 2-870-1 Sakae-Cho Nishi, Matsudo 271-8587, Japan; ^4^Department of Oral Pathology, Nihon University, School of Dentistry at Matsudo, 2-870-1 Sakae-Cho Nishi, Matsudo 271-8587, Japan

## Abstract

Pemphigus Vulgaris (PV) is an autoimmune intraepithelial blistering disease involving the skin and mucous membranes. Oral mucosa is frequently affected in patients with PV, and oral lesions may be the first sign of the disease in majority of patients. In some patients, oral lesions may also be followed by skin involvement. Therefore, timely recognition and therapy of oral lesions is critical as it may prevent skin involvement. Early oral lesions of PV are, however, often regarded as difficult to diagnose, since the initial oral lesions may be relatively nonspecific, manifesting as superficial erosions or ulcerations, and rarely presenting with the formation of intact bullae. Lesions may occur anywhere on the oral mucosa including gingiva; however; desquamtive gingivitis is less common with PV than other mucocutaneous conditions such as pemphigoid or lichen planus. This paper describes the case of a patient presenting with a one-year history of painful gingival, who is finally diagnosed as having PV.

## 1. Introduction

Pemphigus Vulgaris (PV) is an autoimmune intraepithelial blistering disease involving the skin and mucous membranes [1]. PV is characterized by acantholysis in the epithelium [1]. It affects both sexes almost equally and is more common in middle-aged and elderly patients [2, 3]. Systemic corticosteroid therapy is associated with a dramatic improvement of the condition; however, complications of medical therapy remain a concern.

The oral mucous membrane is frequently affected in PV patients; most of patients present with oral lesions as the first sign of PV [4, 5]. Lesions may occur anywhere on the oral mucosa, but the buccal mucosa is the most commonly affected site, followed by involvement of the palatal, lingual, and labial mucosae [2]; the gingiva is the least commonly affected site, and desquamative gingivitis (DG) is a common manifestation of the disease [2].

In many PV patients, the oral lesions are followed by the development of skin lesions [3, 5]. Consequently, if oral PV can be recognized in its early stages, treatment may be initiated to prevent progression of the disease to skin involvement. Early oral lesions of PV are, however, often regarded as difficult to diagnose, since the initial oral lesions may be relatively nonspecific, manifesting as superficial erosions or ulcerations and rarely presenting with the formation of intact bullae [2, 4, 6, 7]. Diagnostic delays of greater than 6 months are common in patients with oral PV [4]. The average interval from the onset to confirmation of the diagnosis of PV has been reported to be 6.8 months [6] or 27.2 weeks [2].

This paper describes the case of a patient presenting with a one-year history of painful gingiva, who was finally diagnosed as having PV. 

## 2. Case Report

A 46-year-old woman was referred to Nihon University School of Dentistry at Matsudo Hospital with a one-year history of painful gingiva. The patient noticed peeling of the gingival epithelium while she brushed her teeth. She had initially received periodontal treatment, including scaling and tooth brushing instructions, from a general dentist; however, she had noted no improvement of the gingival symptom. The oral lesions occurred in repeated cycles of remissions and exacerbations. Oral candidiasis was ruled out by an otologist. 

**Figure 1:**
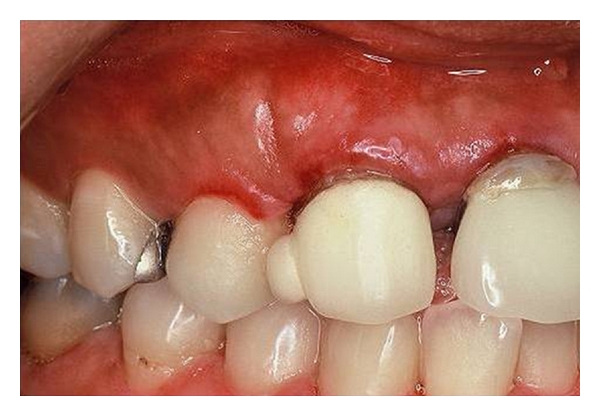
The initial examination revealed a patchy erythematous labial gingiva around teeth no. 7 and 8.

**Figure 2:**
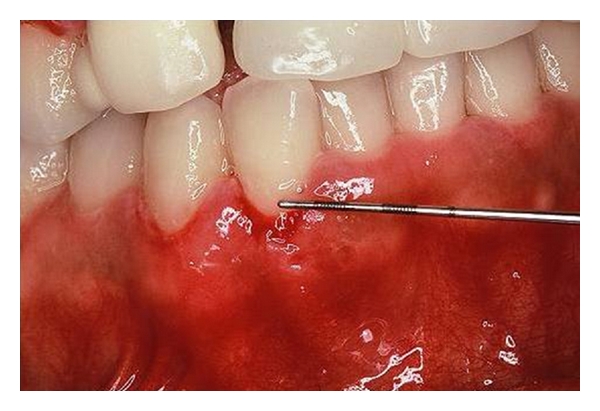
Gentle palpation with a periodontal probe elicited some desquamation of the gingiva around tooth no. 27.

**Figure 3:**
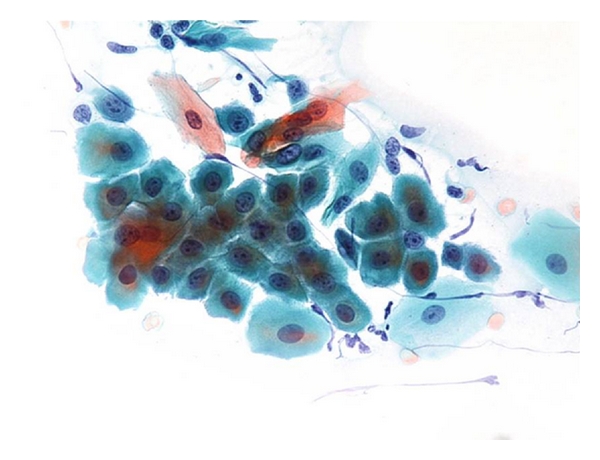
Cytological smear of affected gingiva illustrating a collection of acantholytic Tzank cells.

**Figure 4:**
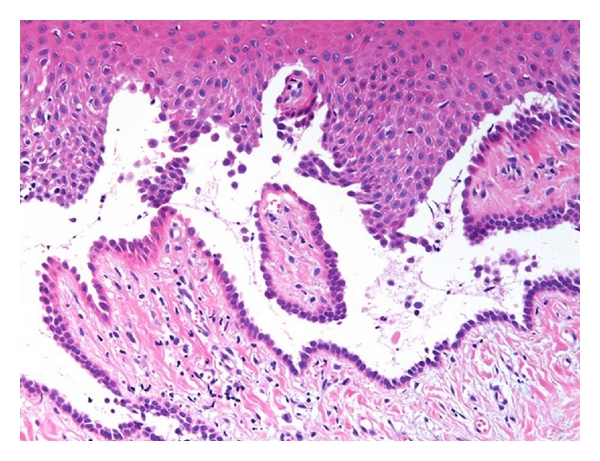
Histopathologic examination of specimens from the gingiva. Suprabasial acantholysis near the tips of two adjacent rete pegs is recognized.

**Figure 5:**
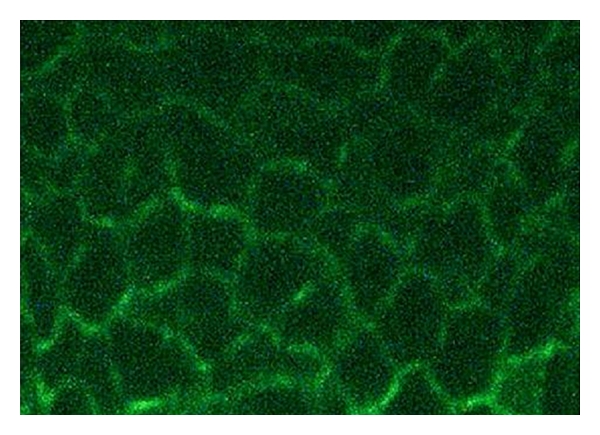
Direct immunofluorescence for deposits of IgG. Deposition of IgG was found between the epithelial cells.

Oral examination revealed localized erosions on the marginal gingiva of teeth no. 7 and 8 (Figure 1). Nikolsky's sign showed a positive reaction, and the epithelium could be peeled away easily by slightly scratching the surface of the gingiva (Figure 2). She had no skin or extraoral lesions, and a review of her medical history was unremarkable. Differential diagnosis included PV, mucous membrane pemphigoid, and erosive lichen planus. The cytological smear was performed before obtaining biopsy specimens. Smears were prepared by exfoliating from the labial gingiva using a cytobrush (Medscand Medical AB, Malmo, Sweden). In the cytological smear, collective acantholytic cells were recognized (Figure 3). These cells enabled a presumptive diagnosis of PV to be made. A gingival biopsy was obtained from the perilesional site and submitted for routine histopathology and the direct immunofluorescence (DIF) test. On histopathological examination, acantholysis was recognized immediately above the basal cell layer (Figure 4). DIF was performed using conjugates for IgG, IgA, IgM, C3, and fibrinogen, and it revealed deposition of IgG and C3 between the epithelial cells (Figure 5). A definitive diagnosis of PV was made based on these clinical and histopathological findings. In this patient, although it took only two weeks from her first visit to our hospital until a definitive diagnosis of PV was made, one year had elapsed from the onset of the oral lesions to the definitive diagnosis.

Topical corticosteroid (0.1% triamcinolone acetonide) was provided for the treatment of gingival lesions. The customized trays were used in order to occlude the topical corticosteroid. The lesions diminished significantly during the four weeks topical corticosteroid therapy. 

## 3. Discussion

 DG is a clinical manifestation of the gingiva that is characterized by desquamation of the gingival epithelium, chronic redness, ulceration, and/or blister formation [5, 7–11]. Nisengard and Levine [10] cited the following as the standard in making a clinical diagnosis of DG: (1) gingival erythema not resulting from plaque, (2) gingival desquamation, (3) other intraoral and sometimes extraoral lesions, and (4) complaint of sore mouth, particularly with spicy foods. It is reported that most cases of DG are caused by several mucocutaneous diseases [5, 7–9].

Mucous membrane pemphigoid and erosive lichen planus are the most frequent causes of DG, accounting for 48.9% and 23.6%, respectively, of all cases of DG [8]. PV is the least common cause of DG (2.3%) [8]. Histopathological examination and DIF testing are necessary to make a definitive diagnosis of the diseases responsible for DG [5, 7, 9–11]. Considering PV is potentially fatal, recognition of gingival lesions of DG, albeit rare, is essential to definitive diagnosis, timely therapy, and followup. In the present patient, it took about one year until a definitive diagnosis of PV was made at our hospital after the first appearance of oral symptoms. During this period, the patient visited a dental clinic, an otorhinolaryngology clinic, and an internal medicine clinic, but definitive diagnosis of PV was not rendered at any of these clinics. The reasons for delayed diagnosis may be explained by: the patient's PV symptoms being confined to the gingiva and being clinically very mild, and the symptoms occurring in repeated cycles of remission and exacerbation.

PV is an autoimmune disease that is characterized by acantholysis in the epithelium [1]. The main antigen in PV is desmoglein (Dsg) 3, a protein constituent of the desmosomes [12]. Most patients with PV have circulating IgG autoantibodies against Dsg3 [12, 13]. These antibodies bind to the Dsg3 on the epithelial cell membrane and may evoke acantholysis [12, 13]. Acantholytic cells are often found in intraepithelial blisters. These cells show degenerative changes, including round, swollen hyperchromatic nuclei with a clear perinuclear halo in cytoplasm. Acantholytic cells can be confirmed in the cytological smear obtained by exfoliating from the oral mucosa [14]. Coscia-Porrazzi et al. [15] showed that acantholytic (Tzanck) cells were recognized in 37 of the 40 PV patients and reported that performing cytomorphologic studies is a useful method to screen the cases suspected to be oral PV. In this report, we recognized acantholytic cells in the cytological smear, which enabled a presumptive diagnosis of PV to be made. However, it is necessary to perform a biopsy since the appearance of acantholytic cells alone does not allow a definitive diagnosis, but only permits a presumptive diagnosis of PV.

This is because acantholytic cells may also appear in other diseases such as impetigo, Darier's disease, transient acantholytic dermatosis, viral infections, and carcinoma [16]. 

For the definitive diagnosis of PV, the following criteria must be fulfilled: (1) the presence of appropriate clinical lesion(s), (2) confirmation of acantholysis in biopsy specimens, and (3) confirmation of autoantibodies in tissue or serum, or both [17]. In the present case, a definitive diagnosis of PV was made based on a general assessment of the following findings: (1) positive Nikolsky's phenomenon, (2) presence of acantholysis in biopsy specimens, and (3) finding of antibody deposition between epithelial cells by DIF test. 

## 4. Conclusion

This report describes the case of a patient presenting with a one-year history of painful gingiva with intractable erosions, who was finally diagnosed as having PV. Although PV is an intraepithelial blistering disease, intact bulla formation of the gingiva is rare and the disease manifests with nonspecific symptoms. Therefore, the diagnosis of PV tends to be delayed. Clinical, histopathological, and immunological examinations should be undertaken to obtain a definitive diagnosis of PV. In patients with PV who have lesions confined to the oral cavity, close followup is essential, and referral to specialists should be undertaken promptly in the event of appearance of extraoral symptoms.
